# Long non-coding RNA NKILA inhibits migration and invasion of non-small cell lung cancer via NF-κB/Snail pathway

**DOI:** 10.1186/s13046-017-0518-0

**Published:** 2017-04-17

**Authors:** Zhiliang Lu, Yuan Li, Jingnan Wang, Yun Che, Shouguo Sun, Jianbing Huang, Zhaoli Chen, Jie He

**Affiliations:** Department of Thoracic Surgery, National Cancer Center/Cancer Hospital, Chinese Academy of Medical Sciences and Peking Union Medical College, Beijing, 10021 China

**Keywords:** Long non-coding RNA, NKILA, Non-small cell lung cancer, Epithelial-mesenchymal transition

## Abstract

**Background:**

Numerous studies have shown that long non-coding RNAs (lncRNAs) play key roles during multiple cancer processes, such as cell proliferation, apoptosis, migration and invasion. The previous studies found that NKILA interacted with and suppressed the nuclear translocation of NF-KappaB, which influenced metastasis and prognosis in breast cancer. However the clinical significance and biological role of NKILA in non-small cell lung cancer (NSCLC) remains unknown.

**Methods:**

We examined expression levels of NKILA in 106 pairs of NSCLC tissues and cell lines. The expression level of NKILA after TGF-β1 stimulation also was examined by qRT-PCR and validated by Chromatin immunoprecipitation (ChIP). Gain-of-function and loss-of-function assays were performed to examine the effect of NKILA on proliferation, migration and invasion of NSCLC cells. RNA immunoprecipitation (RIP), western blot and rescue experiments were carried out to reveal the interrelation between NKILA, NF-κB and EMT signal pathway.

**Results:**

The expression of NKILA was down-regulated in NSCLC cancer tissues compared with matched adjacent noncancerous tissues, and lower NKILA expression in tumor tissues were significantly correlated with lymph node metastasis and advanced TNM stage. We found that the expression of NKILA was mainly regulated by classical TGF-β signal pathway in NSCLC cells rather than NF-κB pathway reported in breast cancer. Gain and loss of function assays found that NKILA inhibited migration, invasion and viability of NSCLC cells. Mechanistic study showed that NKILA attenuated Snail expression via inhibiting the phosphorylation of IκBα and NF-κB activation, subsequently suppressed the expression of markers of epithelial-mesenchymal transition process.

**Conclusions:**

The present study found that the expression of NKILA was downregulated in tumor tissues of NSCLC, which improved the metastasis of NSCLC patients. In vitro studies further clarified that the expression of NKILA was regulated through classical TGF-β signal pathway, which subsequently inhibited migration and invasion of NSCLC cells through interfering NF-κB/Snail signal pathway in NSCLC cells.

**Electronic supplementary material:**

The online version of this article (doi:10.1186/s13046-017-0518-0) contains supplementary material, which is available to authorized users.

## Background

Lung cancer is the most common incident cancer and the leading cause of cancer death all over the world [1]. More than 85% of those cases are currently classified as non-small-cell lung cancer (NSCLC). Although remarkable progress during the past decades in diagnose and treatment, including traditional surgery, chemotherapy, radio-therapy as well as emerging target therapy and immune therapy, the predicted 5-year survival rate is only around 15% - a figure that has only marginally improved during the past few decades [2, 3]. And it is mainly a consequence of regional recurrence and lymph node metastasis. Therefore, a thorough understanding of the molecular mechanism involved in the development and progression of NSCLC could provide more effective diagnostic markers and targets for NSCLC patient therapy.

It is now well known that only less than 2% of the genome encodes proteins, but at least 75% is actively transcribed into noncoding RNAs [4]. Although some of the noncoding transcripts are small, such as miRNA, piRNA and snoRNA, most of them larger than 200 nucleotides in length, which were catalogued as long noncoding RNAs (lncRNAs) [5]. LncRNAs are a highly heterogeneous group of transcripts that regulate gene expression via diverse mechanisms. Consequently, a large amount of lncRNA had been found being dysregulated in a range of caners and contributing to tumorigenesis and tumor progression [6]. For example, the Metastasis-associated lung adenocarcinoma transcript 1 (MALAT1), one of the most highly abundant lncRNAs, was upregulated in NSCLC and correlated with metastasis. The overexpression of MALAT1 can promote the metastasis of lung cancer cells by regulate downstream gene expression and alternative splicing [7]. In contrast, the long non-coding RNA PVT1 suppressed cell growth and induced apoptosis by binding to the enhancer of zeste homolog 2 (EZH2) protein, a histone methyltransferase of the PRC2 complex, in NSCLC [8]. So far, even the exact mechanism of lncRNAs still unknown, most of the lncRNAs exert bio-function via interact with other molecules [9] including mRNA, miRNA, genome DNA, other lncRNAs and mostly proteins, especially proteins in vital signal pathways.

NF-KappaB Interacting LncRNA (NKILA), encoded by a gene at chromosome 20q13 just near by the prostate transmembrane protein androgen induced 1 (PMEPA1) (also named solid tumor associated gene 1, STAG1) [10], is upregulated by NF-κB in breast cancer [11]. Previous study found NKILA can suppress the migration and invasion of breast cancer and tongue squamous cell carcinoma cells via directly binding with NF-κB: IκB complex, thereby inhibiting IKK-induced IκB phosphorylation and NF-κB activation [11, 12]. However, the clinicopathological correlation and functional role of NKILA in NSCLC is still elusive. Moreover, PMEPA1 expression is strongly enhanced by TGF-β and negative feedback inhibit TGF-β/Smad signal pathway in return [13–15], which brings the hypothesis that NKILA may also participate in the TGF-β signal pathway. In this study, we aimed to investigate the role of NKILA in TGF-β pathway, regulating the NF-κB activity and tumor cell EMT in NSCLC, as well as the clinical significance of NKILA in predicting lymph node metastasis.

## Methods

### Tissue samples and clinical data collection

A total of 106 patients with NSCLC who accepted radical surgery therapy in the Department of Thoracic Surgery of Cancer Hospital of the Chinese Academy of Medical Sciences during Nov 2011to Dec2012were enrolled in this study. The pathological diagnosis results for these 106 patients were obtained. None of patients received radiotherapy or chemotherapy before surgery. None of patients was diagnosed as other cancer within 3 years before surgery. TNM stage were classified according to the7^th^ edition of the AJCC lung cancer staging system. Clinicopathological characteristics of these patients were collected. The tumor tissues and adjacent normal tissues of these 106 patients were snap frozen in liquid nitrogen immediately after resection and stored at −80 °C until this study.

### Cell culture and stable cell lines construction

A total of seven cell lines were used including six NSCLC cell lines (H226, H292, H460, A549, ANP973 and H1299) and the immortalized normal human bronchial epithelial cell line BEAS-2B. NSCLC cells were maintained in RPMI-1640 (HyClone) supplemented with 10% FBS (Gibco) and antibiotics (100 U/ml penicillin and 100 mg/ml streptomycin) (Invitrogen). BEAS-2B cells were maintained in BEGM (Lonza). All cell lines were maintained in a humidified air atmosphere at 37 °C with 5% CO_2_. We confirmed cell line identities by matching the short tandem repeat (STR) profile from each line to the registered information in the DSMZ online STR database. TNF-α and IL-1β were purchased from Peprotech and used in 10 ng/ml, TGF-β1were purchased from R&D and used in 5 ng/ml. Times of treatments were 24 h unless specified. To inhibit TGF-β and NF-κB signaling, 10 μM JSH-23 (Selleck) or 5 μM SB505124 (Selleck) were added to culture media 30mins prior to the specified treatments.

Full-length NKILA cDNA was compounded by Generay (Shanghai, China) and ligated into the pCDH-CMV-MCS-EF1-GFP + Puro (CD513B-1) vector. Non-target shRNA control and two shRNAs against NKILA (sh1: 5’-GGA GAA GTC ACA CGT TGA TTG-3’, sh2: 5’-GGC AGT AGG AAA GGA GAA TTG-3’) were obtained from OBiO (shanghai, china). To produce lentivirus containing the NKILA gene or shRNAs target NKILA, HEK-293 T cells were co-transfected with the resulting vector described above, pLP1, pLP2 and pLP/VSVG (Invitrogen) using Lipofectamine 3000 (Life Technologies) strictly according to the manufacturer’s guidelines. Infectious lentiviruses were harvested at 48 h after transfection and filtered through Amicon Ultra-4 Centrifugal Filter Devices (Millipore). A549 and H226 cells were infected with concentrated virus in the presence of 5 μg/ml polybrene (Sigma-Aldrich). The supernatant was replaced with complete culture media after 12 h. The expression of NKILA in the infected cells was confirmed by qRT-PCR 96 h after infection.

### RNA extraction and quantitative real time PCR (qRT-PCR)

The relative RNA levels of genes were assessed by quantitative RT-PCR. In brief, total RNA was isolated with the standard TRIzol-based protocol (Invitrogen), while, cytoplasmic and nuclear RNA were isolated and purified using the Protein and RNA Isolation System (Life technologies) according to the manufacturer’s instructions. RevertAid First-Strand cDNA Synthesis kit (Thermo Scientific) was used for reverse transcription. RT-PCR was performed on an ABI 7900HT Real-Time PCR thermocycler (Life Technologies). Fold differences were calculated according to the 2^−∆∆Ct^ method and normalized against the endogenous expression of GAPDH. The gene-specific primers were as follows: GAPDH (sense: 5’- CCT GGT ATG ACA ACG AAT TTG-3’, antisense: 5’- CAG TGA GGG TCT CTC TCT TCC-3’), NKILA (sense: 5’- AAC CAA ACC TAC CCA CAA CG, antisense: 5’- ACC ACT AAG TCA ATC CCA GGT G-3’), NEAT1 (sense: 5’- GAT GCG CGC CTG GGT GTA GTT-3’, antisense: 5’- CAT GCA GCC TGC CCC ACT GT-3’).

### Cell proliferation and transwell assays

For proliferation assay, treatment and control cells (2 × 10^3^ cells/well) were plated in 96-well plates. Cell viability were measured using the cell counting kit-8 (CCK-8; Dojindo). Cells (5 × 10^4^ or 1 × 10^5^) in serum-free RPMI 1640 medium were plated into the upper chamber of 24-well transwell inserts (Corning, 8.0 μm pores) that were either uncoated or coated with Matrigel (BD Biosciences) for migration or invasion assay. The cells were then allowed to translocate toward medium containing 20% FBS for 24 h. Cells on the lower side of the chamber were fixed, stained and counted in five different areas at 100-fold magnification.

### RNA immunoprecipitation assay (RIP)

A549 cells was stimulated by 5 ng/ml of recombinant TGF-β1 (R&D Systems). After 24 h, cells were used to perform RNA immunoprecipitation (RIP) experiments using an anti-NF-κB antibody (Cell Signaling Technology, CST) and the Magna RIP™ RNA-Binding Protein Immunoprecipitation Kit (Millipore) according to the manufacturer’s instructions. The RNA fraction isolated by RIP was analyzed by qRT-PCR.

### Chromatin immunoprecipitation assay (ChIP)

A549 cells treated with PBS or recombinant TGF-β1 for 30 min were used to perform chromatin immunoprecipitation assay (ChIP) using an anti-Smad2/3 antibody (CST) and the EZ-Magna ChIP Chromatin Immunoprecipitation kit (Millipore) according to manufacturer’s instructions. The chromatin fraction isolated by ChIP was analyzed by qRT-PCR with specific primers for the promoter area of NKILA.

### Western blot analysis

Total cell lysates were prepared in a 1× sodium dodecyl sulfate buffer. Identical quantities of proteins were separated by sodium dodecyl sulfate-polyacrylamide gel electrophoresis and transferred onto nitrocellulose filter membranes. After an incubation with antibodies specific for human GAPDH (CST), p65 (CST), p-p65 (CST), IκBα (CST), p-IκBα (CST), E-cadherin (CST), vimentin (CST), N-cadherin (CST), Snail (CST), Histone 3 (Abcame) or PGEMT (Santa Cruz), the blots were incubated with HRP-conjugated second antibody and were detected using an ImageQuant LAS 4000 (GE).

Subcellular fractions were prepared from A549 and H226 cells with the Protein and RNA Isolation System (Life technologies) according to the manufacturer’s instructions.

### Statistical analysis

Statistical analyses were performed using SPSS version 20.0 (SPSS, Chicago, IL, USA). A Chi-squared test was used to analyze the relationship between NKILA levels and the clinicopathological characteristics. The difference of NKILA expression between groups was evaluated by the Mann-Whitney *U* test. The results of cell experiments were presented as means and S.E.M from three independent experiments, and the differences among groups were analyzed by independent-samples Student’s *t* test. Differences were considered significant at *p* < 0.05.

## Results

### NKILA was downregulated in tumor tissues and correlated with lymph node metastasis of patients with NSCLC

**Fig. 1:**
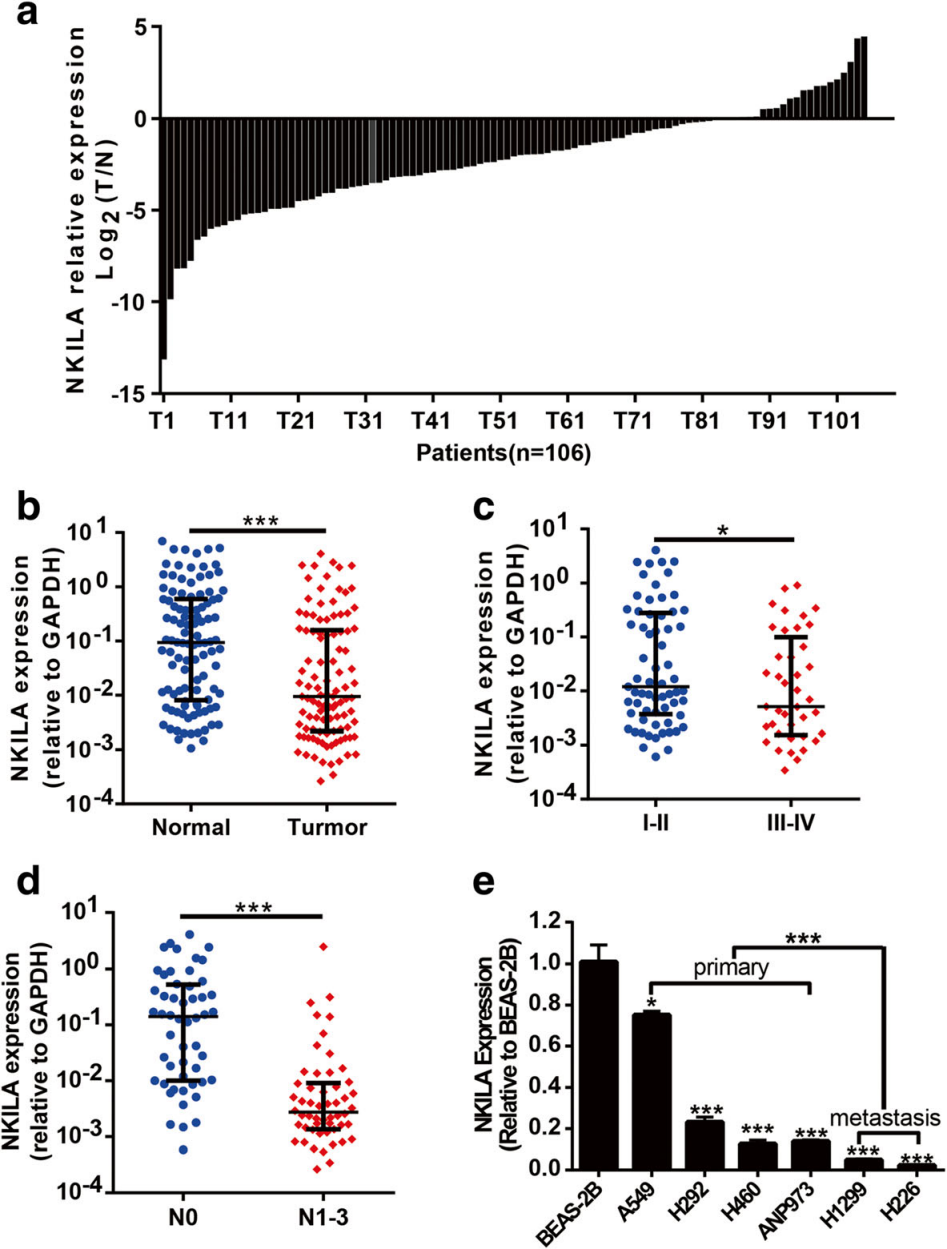
Aberrant Expression of NKILA in NSCLC samples. **a** The fold change of NKILA expression in tumor tissues compared with adjacent normal tissues in 106 patients were shown as log_2_ (Fold change) = log_2_(T_NKILA_/N_NKILA_). Each column represents a patient. **b** The relative NKILA expression (2^-ΔCT^) in 106 tumor tissues was compared with paired adjacent noncancerous lung tissues. **c** The relative expression of NKILA (2^-ΔCT^) was compared between 52 patients without lymph node metastasis (N0) and 54 patients with lymph node metastasis (N1–N3), and **d** between 47patients with advanced stages (III and IV) and 59 patients with early stages (I and II). Median with interquartile range were shown, Mann-Whitney *U* test was used. **e** NKILA expression levels in bronchial epithelial cell line BEAS-2B and six NSCLC cell lines, which are derived from different sites. Two-tailed Student’s *t*-test was used. *p < 0.05, **p < 0.01 and ***p < 0.001

We measured the expression of NKILA in paired tumor tissues and matched adjacent normal tissues from 106 patients with NSCLC using qRT-PCR. The results showed that the expression of NKILA was significantly downregulated in tumor tissues compared with the adjacent normal tissues in these 106 NSCLC patients (*p* < 0.001, Fig. 1a and b).

Next, the relationships between NKILA level and clinicopathological characteristics of patients with NSCLC were analyzed. These 106 patients were classified into high (*n* = 53) or low (*n* = 53) expression of NKILA based on median value of NKILA in tumor tissues. The results showed that the downregulation of NKILA were significantly associated with advanced T stage (*p* = 0.01), larger tumor size (*p* = 0.019), advanced TNM stage (*p* = 0.048, Fig. 1c) and lymph node metastasis (*p* = 0.002, Fig. 1a). In addition, the expression of NKILA was significant downregulated in six NSCLC cell lines compared with human bronchial epithelial cell line BEAS-2B. Furthermore, the expression of NKILA was much lower in NSCLC cell lines derived from metastatic sites than that derived from primary sites (Fig. 1e). All these data suggested that the expression of NKILA was significantly downregulated in NSCLC tumor tissues and implicated in the tumorigenesis and metastasis of NSCLC.

### NKILA inhibited migration, invasion and cell viability of NSCLC cells

**Fig. 2:**
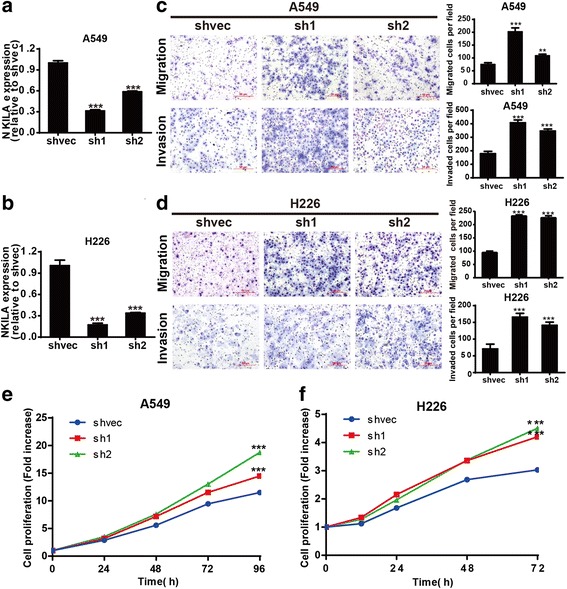
Knockdown of NKILA promote NSCLC cell migration, invasion and proliferation. (**a** and **b**) The stable knockdown efficiency of two specific shRNA against NKILA was examined by qRT-PCR in A549 and H226 cells. (**c** and **d**) The migration and invasion ability of NKILA silenced A549 (**c**) and H226 (**d**) as well as the mock-vehicle control were detected by transwell assay, and images were obtained at 100× magnification. The cell numbers of migration and invasion were compared between groups. (**e** and **f**) Cell proliferation ability were compared between the NKILA shRNA stable transfection and negative control in A549 and H226 cells. Each experiment was performed in triplicate. Data are expressed as means ± SEM. Two-tailed Student’s *t*-test was used. **p < 0.01 and ***p < 0.001

**Fig. 3:**
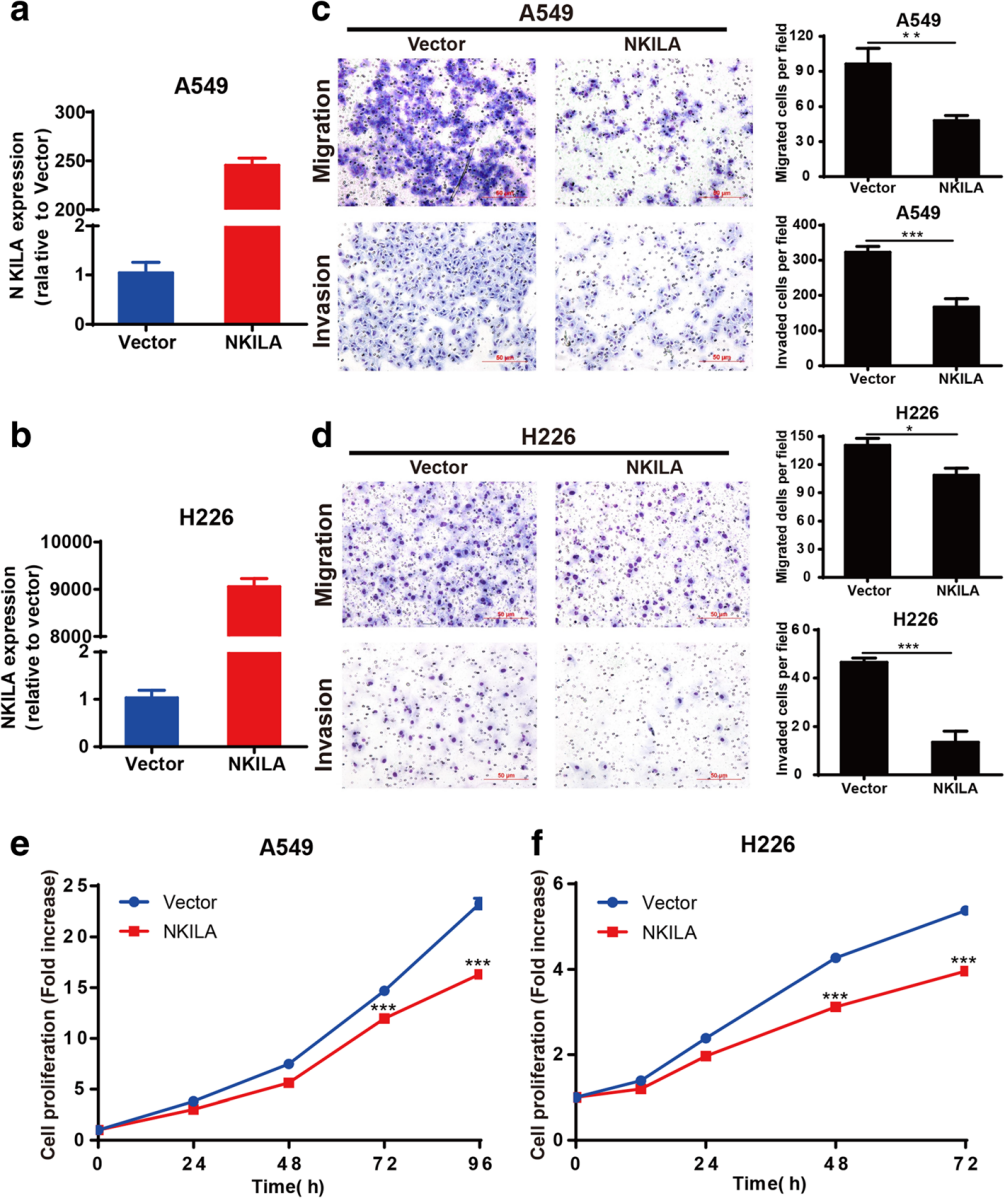
Overexpression of NKILA inhibited NSCLC cell migration, invasion and proliferation. (**a** and **b**) NKILA expression levels of A549 and H226 cells stable transfected with NKILA or mock-vehicle control were examined by qRT-PCR. (**c** and **d**) The migration and invasion ability of NKILA overexpressed A549 (**c**) and H226 (**d**) as well as the mock-vehicle control were detected by transwell assay, and images were obtained at 100× magnification. The cell numbers of migration and invasion were compared between groups. (**e** and **f**) The cell proliferation ability were compared between the NKILA stable transfection and negative control in A549 and H226 cells. Data are expressed as means ± SEM, *n* = 3. Two-tailed Student’s *t*-test was used. *p < 0.05, **p < 0.01 and ***p < 0.001

We further investigated the biological function of NKILA in NSCLC cells. The expression of NKILA was markedly decreased after transfected with two specific NKILA shRNAs (sh1 and sh2) compared with the mock-vehicle control in A549 and H226 cells (Fig. 2a and b). The downregulation of NKILA significantly promoted the migration and invasion ability of A549 and H226 cells compared with the mock-vehicle control (Fig. 2c and d). In addition, compared with the mock-vehicle control, decreased expression of NKILA resulted in a significant increase of cell viability of A549 and H226 cells (Fig. 2e and f). Conversely, the NKILA expression was significantly increased in A549 and H226 cells with stable overexpression of NKILA compared with mock-vehicle control (Fig. 3a and b). The overexpression of NKILA significantly suppressed migration and invasion ability (Fig. 3c and d) and also decreased cell viability of A549 and H226 cells (Fig. 3e and f). These results showed that NKILA negatively regulated migration, invasion and viability of NSCLC cells.

### The expression of NKILA was regulated through TGF-β signal pathway in NSCLC cells

**Fig. 4:**
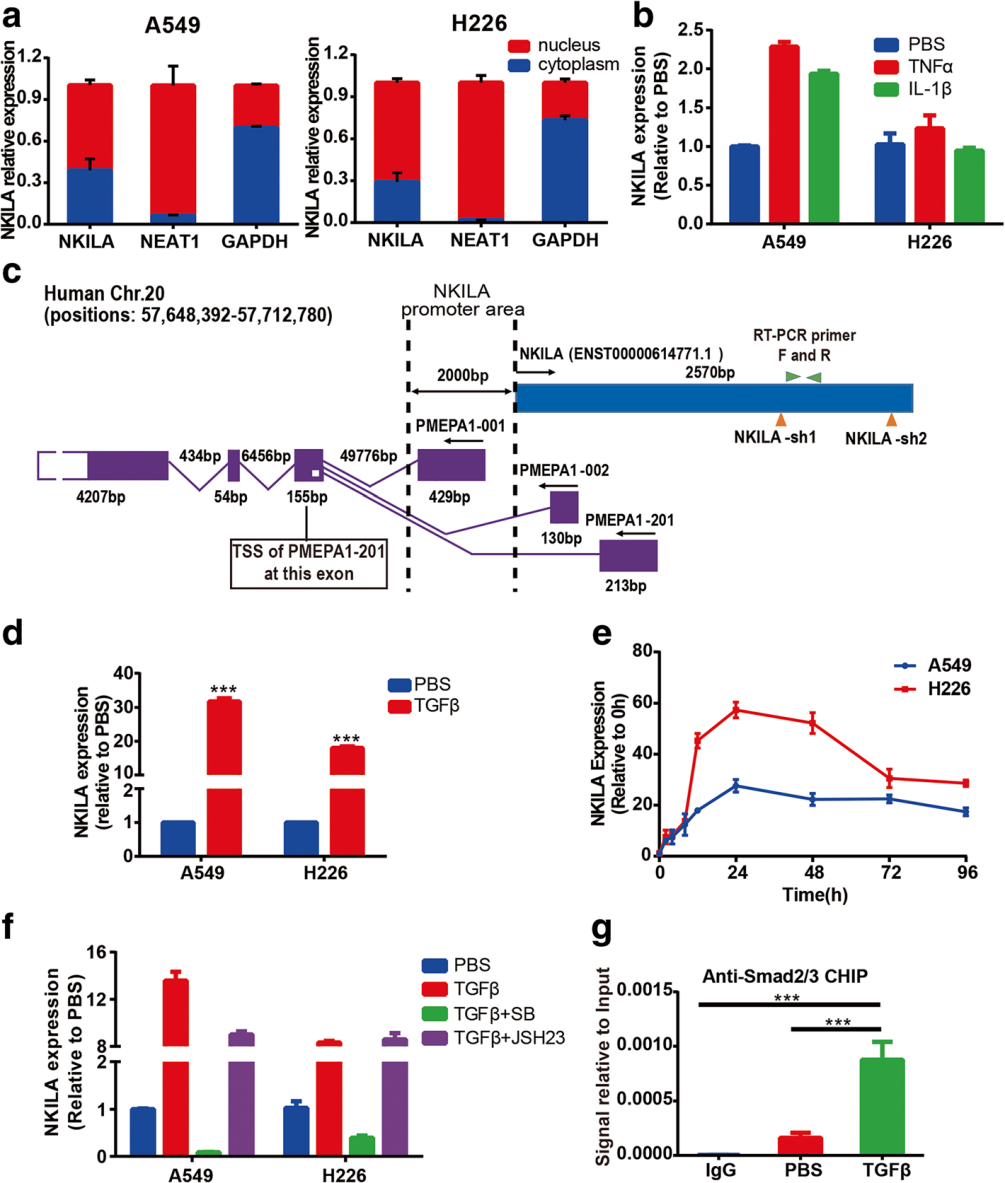
Regulation of NKILA expression in NSCLC cells. (**a**) qRT-PCR was performed to detect the relative NKILA levels in A549 and H226 cell cytoplasm and nucleus. GAPDH and NEAT1 RNA were used as fractionation indicators. (**b**) NKILA expression levels of A549 and H226 treated with TNFα, IL-1β or PBS were detected by qRT-PCR. (**c**) The schematic diagram of PMEPA1 and NKILA genome location. Exons are indicated by boxes, arrows indicate the direction of transcription. (**d**) The NKILA expression levels of A549 and H226 treated with TGF-β1 or PBS were detected by qRT-PCR. (**e**) NKILA expression kinetics in A549 and H226 cells following TGF-β1 stimulation. (**f**) Expression of NKILA assayed by qRT-PCR in A549 and H226 cells induced by TGF-β1 with or without TGF-β or NF-κB inhibition by SB505124 (SB) and JSH-23 (JSH) respectively. (**g**) Localization of Smad2/3 complex to NKILA promoter in A549 cells treated with TGF-β1 or PBS for 30 mins analyzed by ChIP. Data are expressed as means ± SEM, *n* = 3. Two-tailed Student’s *t*-test was used. ***p < 0.001

To figure out the cellular localization of NKILA in NSCLC cells, we isolated total RNA from nuclear and cytosolic fractions of A549 and H226 cells and measured NKILA expression in both fraction by qRT-PCR. Nucleic RNA NEAT1 was chosen as positive control. The result showed that the expression of NKILA was located in nucleus and cytoplasm simultaneously, and the NKILA level was slightly higher in nucleus than in cytoplasm in A549 and H226 cells (Fig. 4a).

Previous study found that the expression of NKILA was regulated by TNF-α or IL1β induced NF-κB activation in breast cancer cells [11]. However, in our study, TNF-α or IL1β treatment led to about only 2-fold increase of NKILA expression in A549 cells, and no increase in H226 cells compared with PBS (Fig. 4b). In view of the genomic location of NKILA just next to PMEPA1 (Fig. 4c), and the transcription of PMEPA1 was reported to be regulated by TGF-β signal pathway [14, 15], which was also verified in our study in A549 and H226 cells (Additional file 1: Figure S1). Moreover, there are a couple of smad2/3 binding motif (Additional file 2: Table S1) at the promoter area of NKILA predicted by JASPAR (http://jaspar.genereg.net/). Therefore, we hypothesized that the transcription of NKILA was regulated by TGF-β signal pathway in NSCLC cells. The expression of NKILA were detected after treatment with TGF-β1 for 24 h in A549 and H226 cells. The results showed that TGF-β1 led to dramatically increase of NKILA transcription in A549 and H226 cells (Fig. 4d). And NKILA expression peaked at 24 h after TGF-β1 treatment and remained high level for up to 96 h in A549 and H226 cells (Fig. 4e). Next, TGF-β receptor inhibitor SB505124 was used to abrogate the effect of TGF-β1 treatment, and the result showed that SB505124 significantly abrogate the TGF-β1-induced NKILA expression in A549 and H226 cells (Fig. 4f). These results proved that the expression of NKILA was mainly regulated by TGF-β1 in NSCLC cells.

To further investigate whether NKILA is regulated by classical TGF-β pathway, the ChIP assay was performed using anti-Smad2/3 antibody. The result showed that TGF-β1 treatment led to significant increase of NKILA promoter sequence, which implied Smad2/3 complex being recruited to the promoter of NKILA gene through TGF-β1 treatment (Fig. 4g). Moreover, we then investigated whether TGF-β signaling is responsible for the induced NKILA transcription. The NF-κB nuclear translocation inhibitor JSH-23 was used to abrogate the activation of NF-κB signal pathway. The results showed that JSH-23 partially abrogated the effect of TGF-β1 in A549 cells and no decrease of NKILA was found in H226 cells (Fig. 4f). These results indicated that the transcription of NKILA was upregulated mainly by classical TGF-β signaling pathway in NSCLC cells.

### NKILA inhibited IκBα phosphorylation and NF-κB activation in NSCLC cells

**Fig. 5:**
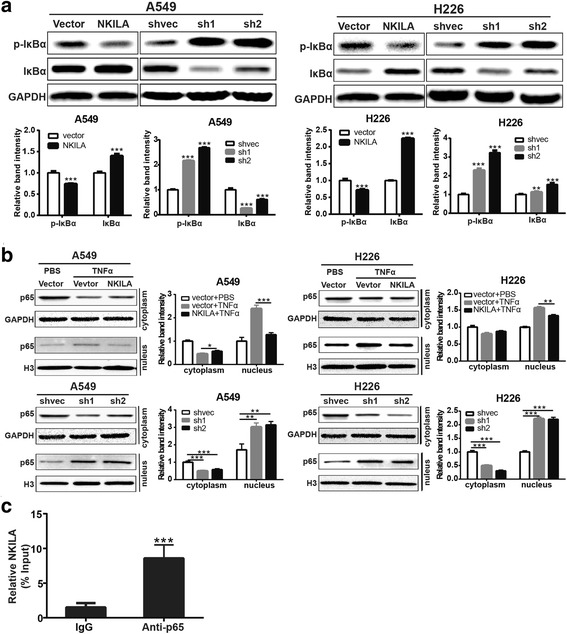
NKILA negatively regulate IκB phosphorylation and NF-κB activation. (**a**) Western blotting showing total and phosphorylated IκBα in A549 and H226 cells. Left panel was representative images and *right panel* was statistical column diagram. (**b**) Western blot for nuclear and cytoplasm p65 in A549 and H226 cells. GAPDH and Histone 3 (H3) is the loading control for cytoplasm and nuclear, respectively. Left panel was representative images and *right panel* was statistical column diagram. (**c**) RIP-derived RNA was measured by qRT-PCR. The levels of qRT-PCR products were expressed as a percentage of input RNA. Data are expressed as means ± SEM. Two-tailed Student’s *t*-test was used. *p < 0.05, **p < 0.01, ***p < 0.001

It was reported that NKILA interacted with and influenced the activation of NF-κB in breast cancers [11]. In present study, the effect of NKILA on IκBα phosphorylation level was detected in A549 and H226 cells. TNF-α (20 ng/ml) was used to induce the NF-κB activation in A549 and H226 cells. The results showed that overexpression of NKILA led to significant decrease of phosphorylation level of IκBα in A549 and H226 cells. Whereas, knockdown expression of NKILA resulted in significant increase of phosphorylation level of IκBα (Fig. 5a). Furthermore, we detected the effect of NKILA on the activation of NF-κB. The results showed that overexpression of NKILA led to significant cytoplasm retention of p65 after TNF-α treatment compared with mock-vehicle control, Whereas, knockdown expression of NKILA significantly improved the nuclear translocation of p65 after TNF-α treatment (Fig. 5b). Moreover, RNA immunoprecipitation was performed with anti-p65 antibody in A549 cells. The result showed that NKILA was significantly enriched by anti-p65 antibody compared to IgG control in A549 cells (Fig. 5c). These results indicated that NKILA inhibited phosphorylation of IκBα and p65 nuclear translocation through interacting with NF-κB in NSCLC cells.

### NKILA inhibited migration of NSCLC cells through NF-κB/Snail signal pathway

**Fig. 6:**
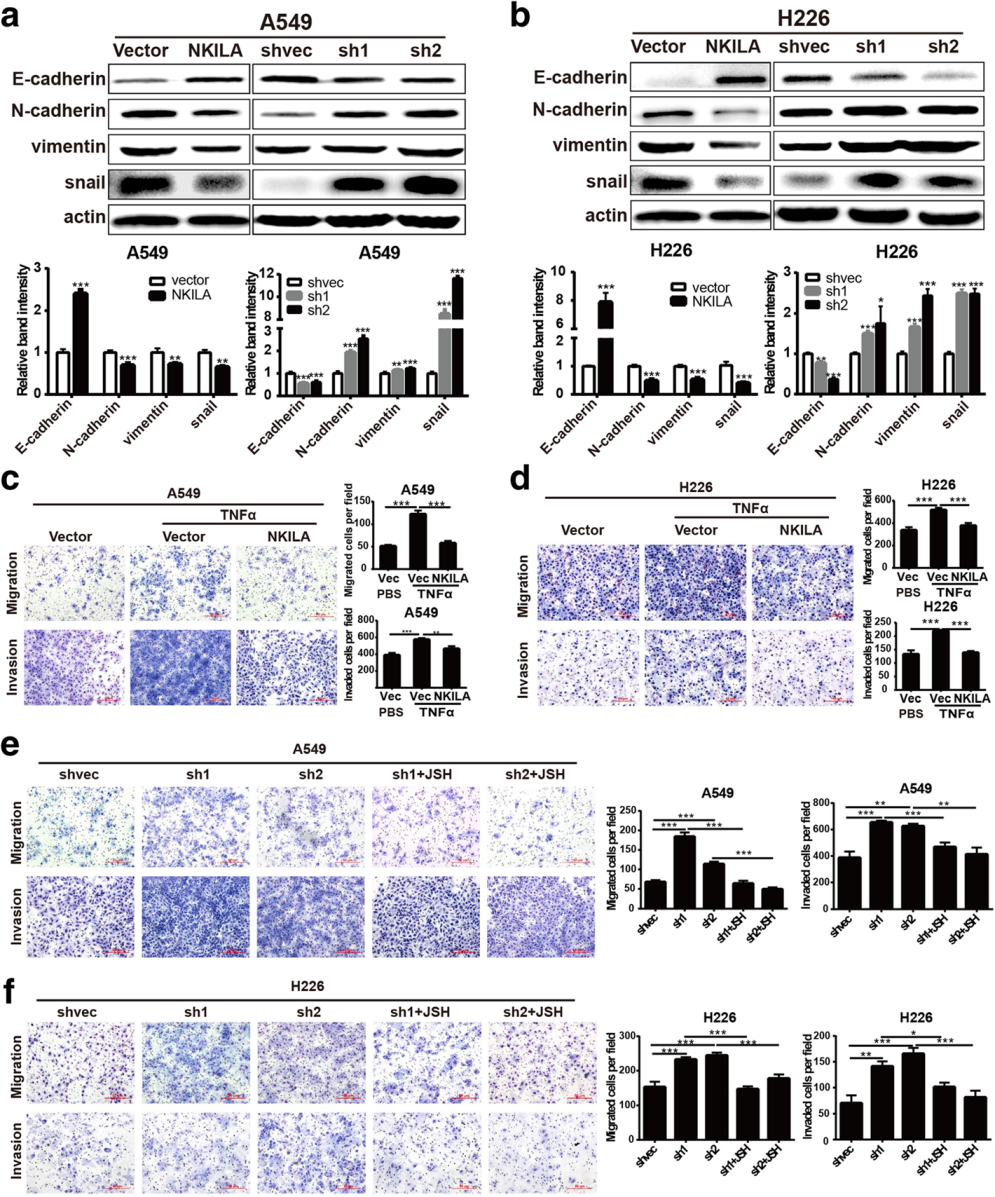
NKILA regulate NSCLC cell mobility via NF-κB/Snail pathway. (**a** and **b**) The expression levels of classical EMT markers in A549 (**a**) and H226 (**b**) cells were measured by western blot. (**c** and **d**) Migration and invasion ofA549 and H226 stably expressing NKILA or mock-vehicle control with or without TNFα stimuli measured by Chamber assay. (**e** and **f**) Migration and invasion of A549 and H226 stably expressing NKILA shRNA or negative control with or without JSH-23 (JSH) measured by Chamber assay. Left panel was representative images and *right panel* was statistical column diagram. Data are expressed as means ± SEM, *n* = 3. *p < 0.05, **p < 0.01, ***p < 0.001

EMT, the key downstream of TGF-β signaling, is one of the most important signal pathway to determine cell metastasis capability, and has abundant crosstalk with NF-κB signal pathway [16]. The above results indicated that the expression of NKILA was regulated by TGF-β signal pathway, and NKILA subsequently inhibited the NF-κB activation. Therefore, we hypothesized that NKILA influenced EMT process of NSCLC cells. Firstly, the classical markers of EMT were detected by western blot. The results showed that the overexpression of NKILA led to significant increase of E-cadherin, and decrease of N-cadherin, vimentin and snail in A549 and H226 cells compared with mock-vehicle control. Whereas, the knockdown of NKILA led to obvious decrease of E-cadherin accompanied by the elevated expression of N-cadherin, vimentin and snail in A549 and H226 cells (Fig. 6a and b). Moreover the effects of NKILA on the expression of EMT markers can be abrogated by NF-κB translocation inhibitor JSH-23 (Additional file 1: Figure S2). To further evaluate whether NF-κB/Snail pathway is responsible for cell migration and invasion regulated by NKILA. TNF-α was used to induce the activation of NF-κB, and subsequently promoted migration and invasion ability of A549 and H226 cells. The results showed that the overexpression of NKILA reversed the effect of TNF-α on migration and invasion (Fig. 6c and d). Whereas, the knockdown of NKILA significantly improved the migration and invasion capacity of A549 and H226 cells, which was markedly abrogated by NF-κB translocation inhibitor JSH-23 (Fig. 6e and f). Collectively, all these results suggested that NKILA regulated migration and invasion of NSCLC cells through NF-κB/Snail pathway.

## Discussion

With the development of next-generation sequencing and applying to a growing number of cancer transcriptomes has indeed revealed thousands of lncRNAs whose aberrant expression is associated with different cancer types. Among the few that have been functionally characterized, a considerable number of lncRNAs have key roles in tumor signal pathway regulation and thus affect various aspects of tumorigenesis and progression, including proliferation, survival, migration or genomic stability [17]. Unlike mRNAs, lncRNAs are more often conserved of short sequence motifs or secondary structure and act as a molecular scaffolds that interact with multiple regulatory proteins [9]. For example, it was reported that NRON, as a molecular scaffold, is necessary for the assembly of NFAT and several NFAT kinases, which are responsible for NFAT phosphorylation and its sequestration in the cytosol [18]. In contrast, NKILA binding with NF-κB: IκB complex and masking the phosphorylation sites of IκB from IKK to inhibit the IKK-induced IκB phosphorylation [11], which was indirectly verified in NSCLC via RIP assay. Consistent with previous research, we further found overexpression of NKILA can inhibit the phosphorylation of IκB and NF-κB translocation to nucleus in NSCLC cells.

As a transcription factors, NF-kB regulates many downstream genes expression including that regulate cell metastasis and plays pivotal roles in both promoting and maintaining an invasive phenotype. In our study we found increasing NKILA expression in NSCLC cells can significantly inhibit cell migration invasion and proliferation. Silencing NKILA expression in A549 and H226 obviously increased the cell viability, migration and invasion ability. However, these phenotypes can be reversed by NF-kB inhibitor. These results suggested that NF-κB is responsible for NKILA-regulated malignant phenotype.

In mammalian cells, Snail has been shown to be a direct repressor of E-cadherin gene transcription though specific E-boxes in the proximal promoter [16] as well as upregulate the expression of N-cadherin and vimentin [19], which are the hollmarks of EMT. As a critical regulator of EMT signaling pathways, overexpression of Snail correlates with tumor grade, nodal metastasis, and tumor recurrence and predicts a poor outcome in patients with various cancers [20–25]. The expression of Snail can be regulated by NF-κB through both transcriptional and post-translational mechanisms [26]. On the one hand, NF-κB can bind to the human Snail promoter and increase Snail transcription directly [27]. On the other hand, NF-κB can blocks the ubiquitination and degradation of Snail, which is required for inflammation-induced cell migration and invasion [28]. Knockdown of Snail expression inhibited cell migration and invasion induced by inflammation, which mainly owe to NF-κB [28]. In lung adenocarcinoma the TGF-β induced EMT can be inhibited by suppressing NF-κB mediated Snail activation [29]. All these finding can demonstrates that Snail plays fundamental role in NF-κB-regulated migration, invasion, and metastasis. In our study we also found when overexpressed NKILA, which attenuated the NF-κB activation, the Snail expression was decreased accompany with increase of E-cadherin level and decrease of N-cadherin and Vimentin, as well as weakened migration and invasion ability. Contrarily, knockdown of NKILA increased the expression of snail followed with attenuation of E-cadherin and enhanced N-cadherin and vimentin expression, which obviously increased the malignant phenotype of NSCLC cells. What’s more the influence on Snail, EMT markers and cell mobility caused by NKILA can be abolished by NF-κB inhibitor. Collectively, we can draw a conclusion that the NKILA regulated migration and invasion is mediated by NF-κB/Snail signal pathway.

TGF-β and NF-κB, two vital signal pathway in cancer, have complex interaction between each other. TGF-β can active NF-κB signal pathway via phosphorylating IKK2 and NIK [30], on the other hand NF-κB mediated Smad7 induction inhibits Smad2/3 activation [31]. LncRNA, as an emerged crucial regulators of various biological processes, present strong ability to link different signal pathway together, and supplement the signal network of cancer [32]. For example, the TGF-β1-upregulated lncRNA malat1 can active PI3K/AKT pathway and promote the proliferation and invasion of human osteosarcoma [33–35]. For the genome location of NKILA just besides PMEPA1, which has been proven being upregulated by TGF-β [15, 36],and play an important role in NSCLC [37, 38]. In our study, we found NKILA can be dramatically upregulated by TGF-β1 treatment in NSCLC cells. However, contrary to earlier reports in breast cancer, just no more than 2-fold increase following TNF-α or IL1β treatment. What’s more, the TGF-β1 induced NKILA expression can’t be reversed by NF-κB nuclear translocation inhibitor JSH-23, while, TNF-α or IL1β induced NKILA upregulation can be absolutely abrogated by TGF-β receptor inhibitor SB505124 (Additional file 1: Figure S3). Combining with the ChIP assay result shown in Fig. 4g we could draw the conclusion that NKILA is directly upregulated by TGF-β classical pathway, however, the NKILA expression alter brought by NF-κB pathway may indirectly through TGF-β signaling in NSCLC cells. Subsequently, increased NKILA expression can inhibit the activation of NF-κB and then suppress the expression of Snail, which is a critical role upregulated by TGF-β signal pathway. Our study provides a new plausible interaction between TGF-β and NF-κB signal pathway.

TGF-β pathway plays dual anti- and pro-tumoral roles in a large panel of cancer types including NSCLC. In early-stage tumors, the TGF-β classical pathway have relative high activation and prevents incipient tumorigenesis [39]. While, with accumulation of genetic alterations of the core components and tumor-suppressive arm of TGF-β pathway lead it converse from tumor-suppressive to tumor-promoting activities [40, 41]. In our study, we found NKILA was upregulated by classical TGF-β pathway and had higher expression level in early stages (TNM stageIandII) than advanced stages (TNM stage III and IV) of NSCLC (*p* = 0.048, Table 1), which was consistent with the dual roles of TGF-β in cancer. Even exact mechanism is still unclear, NKILA brought further understanding of the contextual pleiotropy of TGF-β pathway in NSCLC.

**Table 1:** Correlation between the clinicopathologic features and expression of NKILA

Characteristics	NKILA relative expression *N* (%)		*p*
	Low expression (53)	High expression (53)	
Age (y)			
< 60	27 (48.2)	29 (51.8)	0.697
≥ 60	26 (52.0)	24 (48.0)	
Gender			
Male	36 (49.3)	37 (50.7)	0.834
Female	17 (51.5)	16 (48.5)	
Differentiated degree			
Well	1 (1.9)	5 (9.4)	0.212
Media	33 (62.3)	28 (52.8)	
Poor	19 (35.8)	20 (37.7)	
Pathological type			
Squamous carcinoma	26 (49.1)	27 (50.9)	0.846
Adenocarcinoma	27 (50.9)	26 (49.1)	
Clinical stage			
I-II	25 (44.1)	34 (55.9)	**0.048** ^*^
III-IV	29 (57.4)	18 (42.6)	
T stage			
I-II	36 (43.4)	47 (56.6)	**0.010** ^*^
III-IV	17 (73.9)	6 (16.1)	
Tumor size			
< 3 cm	10 (32.3)	21 (67.7)	**0.019** ^*^
≥ 3 cm	43 (57.3)	32 (42.7)	
Lymph node metastasis			
Negative	18 (34.6)	34 (63.4)	**0.002** ^*^
Positive	35 (64.8)	19 (35.2)	

*Bolded data indicates statistically significant differences

Finally, our findings have potential clinical application value. In recent years, lncRNAs has been attracted more and more attention as potential therapeutic target for cancer. Arun and colleagues provided an exciting example of the potential of noncoding RNAs as therapeutic targets in the treatment of breast cancer [42]. Almost all of the lncRNAs exert function via special secondary structure and functional motif, which can be artificially synthesized. Therefore, engineered ncRNAs containing the particular functional domains have potential to be used for therapy. The expression of NKILA is negatively correlated with NSCLC lymph node metastasis suggest NKILA could be an effective marker and target for antimetastasis therapies.

## Conclusions

**Fig. 7:**
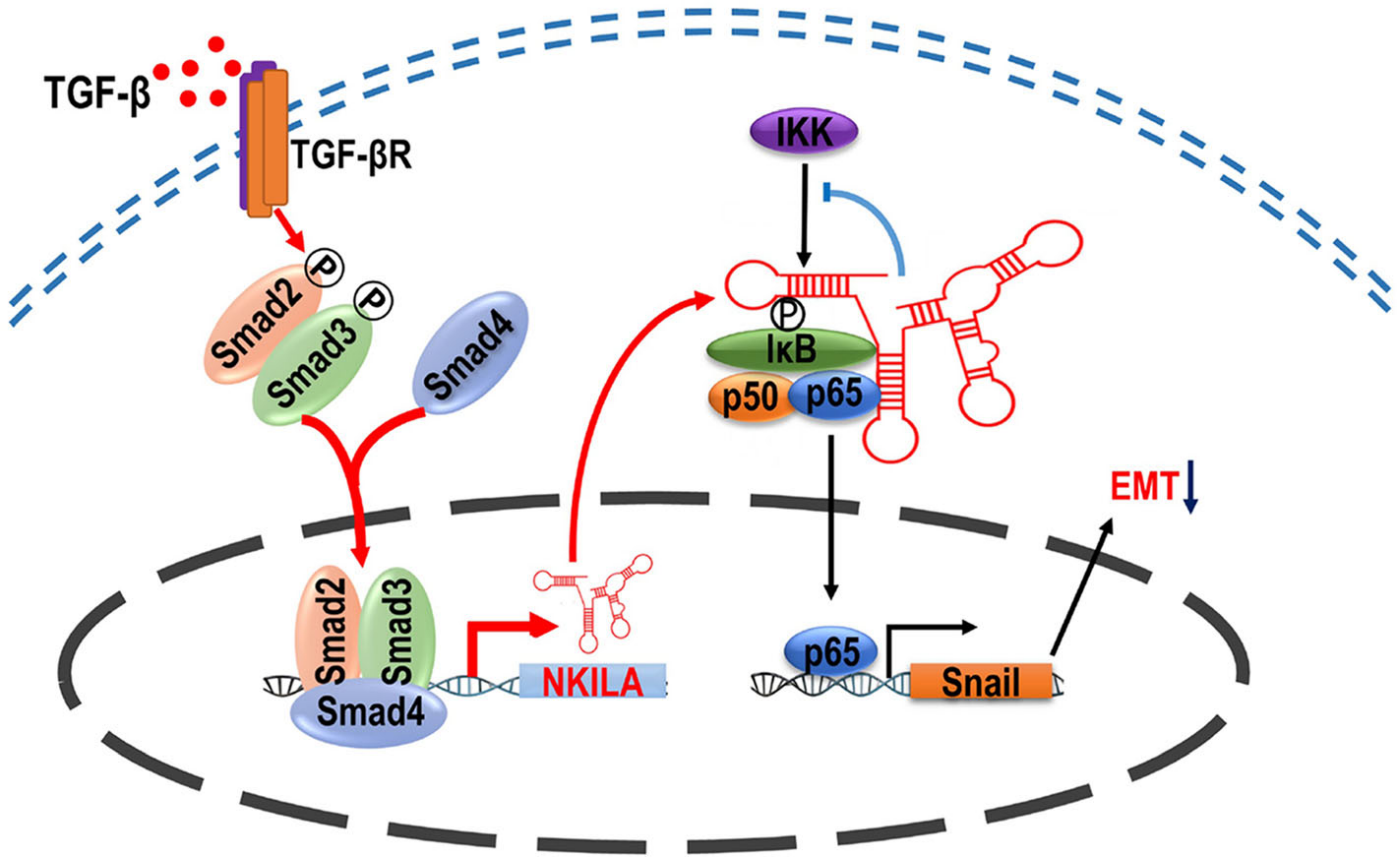
Schematic model of *NKILA* functions during the invasion-metastasis cascade in NSCLC. NKILA, which could be directly activated by TGF-β, inhibits NSCLC cell migration and invasion by binding the *NF-κB: IκB* complex and masking the phosphorylation sites of *IκB* from *IKK* to inhibit the IKK-induced *IκB* phosphorylation and *NF-κB* activation. Subdued *NF-κB* signal pathway activation lead to lower snail expression and then suppress *NSCLC* cell *EMT*

In summary, NKILA is activated by TGF-β and inhibits NSCLC cell migration and invasion by inhibiting IκB phosphorylation and NF-κB activation then suppressing Snail regulated EMT (Fig. 7). Furthermore, NKILA expression is negatively associated with tumor metastasis in patients with NSCLC. These observations provide a potential therapeutic target for metastasis of NSCLC.

## Additional files

Additional file 1: Figure S1. PMEPA1 was upregulated by TGF-β1. Western blot for PMEPA1 in A549 and H226 cells with or without TGF-β1 induce. GAPDH is the loading control. **Figure S2.** NKILA-regulated Snail/EMT pathway change can be abrogated by NF-κB inhibitor JSH-23. NKILA-knockdown cells were incubated in TNFα with or without NF-κB inhibitor JSH-23, and the expression levels of classical EMT markers and p-IκB were measured by western blot. GAPDH is the loading control. **Figure S3.** NF-κB regulated NKILA expression can be reversed by TGF-β inhibitor. NKILA expression levels of A549 (A) and H226 (B) treated with TGF-β1 with or without JSH-23 (JSH) as well as the NKILA expression levels of NSCLC cells treated with TNFα or IL1β with or without SB505124 (SB) were detected by qRT-PCR. Data are expressed as means ± SEM, *n* = 3. Two-tailed Student’s *t*-test was used. *p < 0.05, ***p < 0.001, ns means no statistical significance. (DOCX 566 kb)

Additional file 2: Table S1. JASPAR predicted Smad2/3 binding sites of NKILA promoter area. (DOCX 14 kb)

## Abbreviations

CCK-8: Cell counting kit-8
ChIP: Chromatin immunoprecipitation
EMT: Epithelial-mesenchymal transition
FBS: Fetal bovine serum
NSCLC: Non-small cell lung cancer
PBS: Phosphate Buffered Saline
RIP: RNA immunoprecipitation
STR: Short tandem repeat
