# Correction: Long non-coding RNA NKILA inhibits migration and invasion of non-small cell lung cancer via NF-κB/Snail pathway

**DOI:** 10.1186/s13046-025-03410-x

**Published:** 2025-05-19

**Authors:** Zhiliang Lu, Yuan Li, Jingnan Wang, Yun Che, Shouguo Sun, Jianbing Huang, Zhaoli Chen, Jie He

**Affiliations:** Department of Thoracic Surgery, National Cancer Center/Cancer Hospital, Chinese Academy of Medical Sciences and Peking Union Medical College, Beijing, 10021 China

**Correction: J Exp Clin Cancer Res 36, 54 (2017)**

**https://doi.org/10.1186/s13046-017–0518-0**

Following the publication of the original article [1], the authors found errors in the published version of Fig. 5 and Fig. 6, specifically:

1. In Fig. 5a on page 8, the internal reference of Western Blot should be actin and not GAPDH;

2. In Fig. 5a on page 8 and Fig. 6b on page 9, the internal reference bands of H226 NKILA overexpression were accidentally used duplicate images during editing;

3. The same graphs were used between the H226-shvec invasion and H226-sh2 invasion in Fig. 2d and Fig. 6f this is because the authors conducted the experiments at the same time and thought that it was reasonable using the same graphs to display the same cell line with receiving the same treatment. However, in order to avoid unnecessary misunderstandings, the authors think it is more reasonable to present the graphs of different batches from repeated experiments. Thence, the graphs of H226-shvec invasion and H226-sh2 invasion in Fig. 6f should be replaced.

Below are the correct figures:

Incorrect Fig. 5

**Fig. 5:**
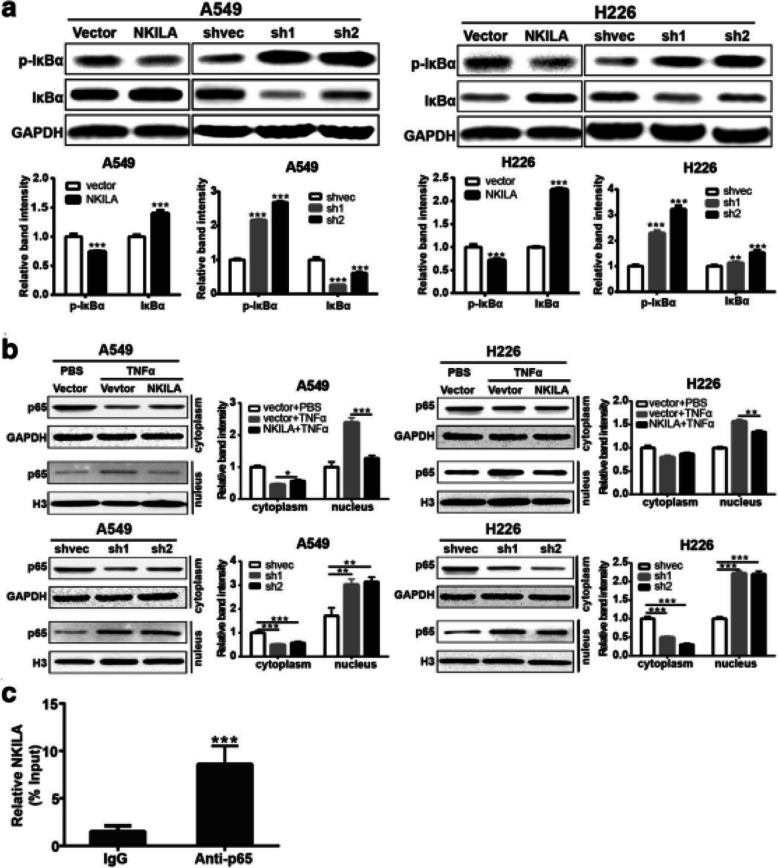
NKILA negatively regulate IκB phosphorylation and NF-κB activation. **a** Western blotting showing total and phosphorylated IκBα in A549 and H226 cells. Left panel was representative images and *right panel* was statistical column diagram. **b** Western blot for nuclear and cytoplasm p65 in A549 and H226 cells. GAPDH and Histone 3 (H3) is the loading control for cytoplasm and nuclear, respectively. Left panel was representative images and *right panel* was statistical column diagram. **c** RIP-derived RNA was measured by qRT-PCR. The levels of qRT-PCR products were expressed as a percentage of input RNA. Data are expressed as means ± SEM. Two-tailed Student’s *t*-test was used. *p < 0.05, **p < 0.01, ***p < 0.001

Correct Fig. 5

**Fig. 5:**
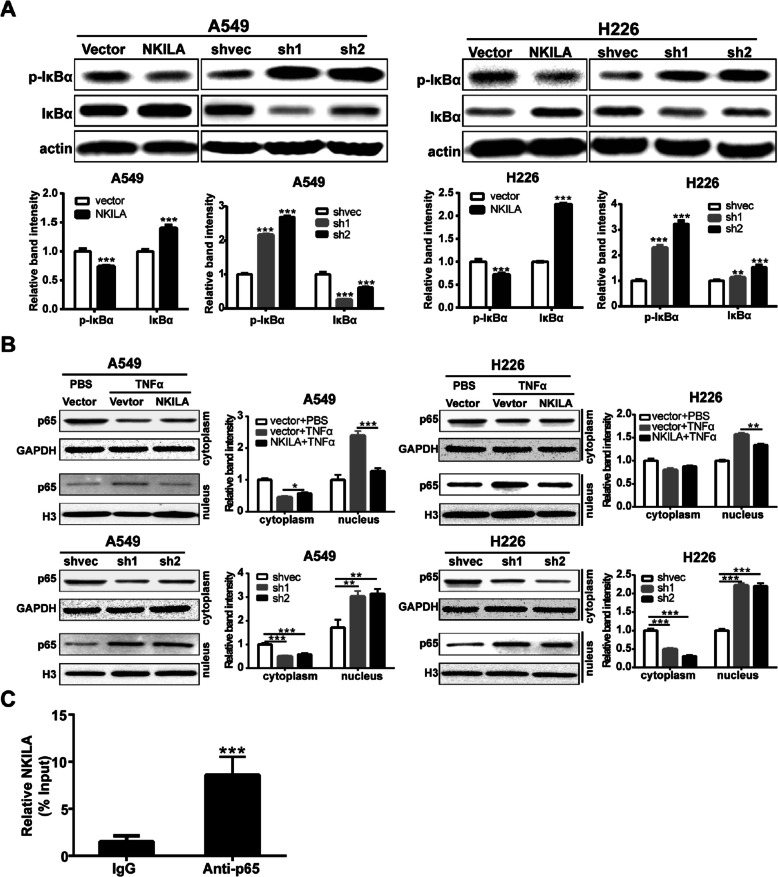
NKILA negatively regulate IκB phosphorylation and NF-κB activation. **a** Western blotting showing total and phosphorylated IκBα in A549 and H226 cells. Left panel was representative images and *right panel* was statistical column diagram. **b** Western blot for nuclear and cytoplasm p65 in A549 and H226 cells. GAPDH and Histone 3 (H3) is the loading control for cytoplasm and nuclear, respectively. Left panel was representative images and *right panel* was statistical column diagram. **c** RIP-derived RNA was measured by qRT-PCR. The levels of qRT-PCR products were expressed as a percentage of input RNA. Data are expressed as means ± SEM. Two-tailed Student’s *t*-test was used. *p < 0.05, **p < 0.01, ***p < 0.001

Incorrect Fig. 6

**Fig. 6:**
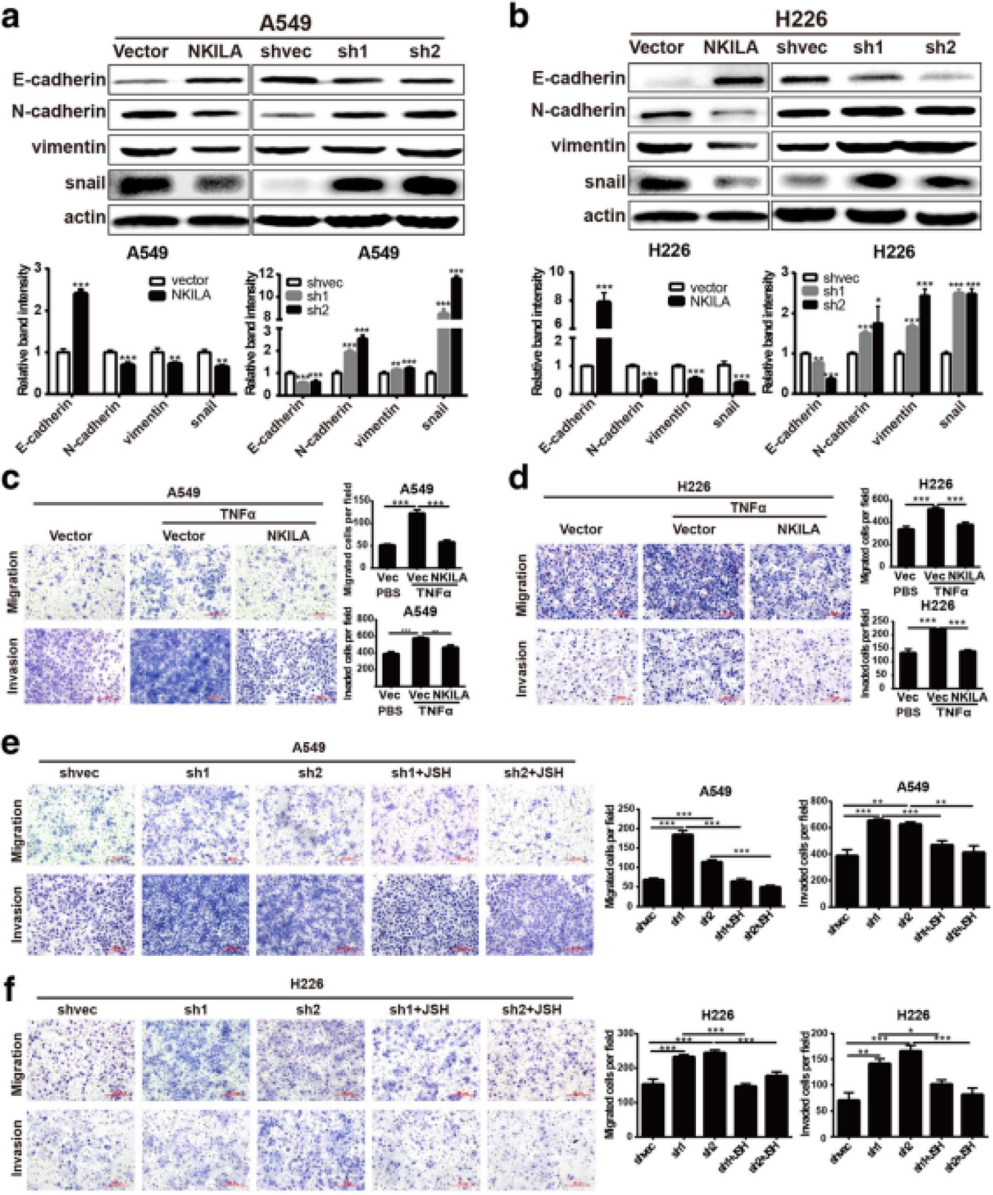
NKILA regulate NSCLC cell mobility via NF-κB/Snail pathway. **a** and **b** The expression levels of classical EMT markers in A549 (**a**) and H226 (**b**) cells were measured by western blot. **c** and **d** Migration and invasion ofA549 and H226 stably expressing NKILA or mock-vehicle control with or without TNFα stimuli measured by Chamber assay. (**e** and **f**) Migration and invasion of A549 and H226 stably expressing NKILA shRNA or negative control with or without JSH-23 (JSH) measured by Chamber assay. Left panel was representative images and *right panel* was statistical column diagram. Data are expressed as means ± SEM, *n* = 3. *p < 0.05, **p < 0.01, ***p < 0.001

Correct Fig. 6

**Fig. 6:**
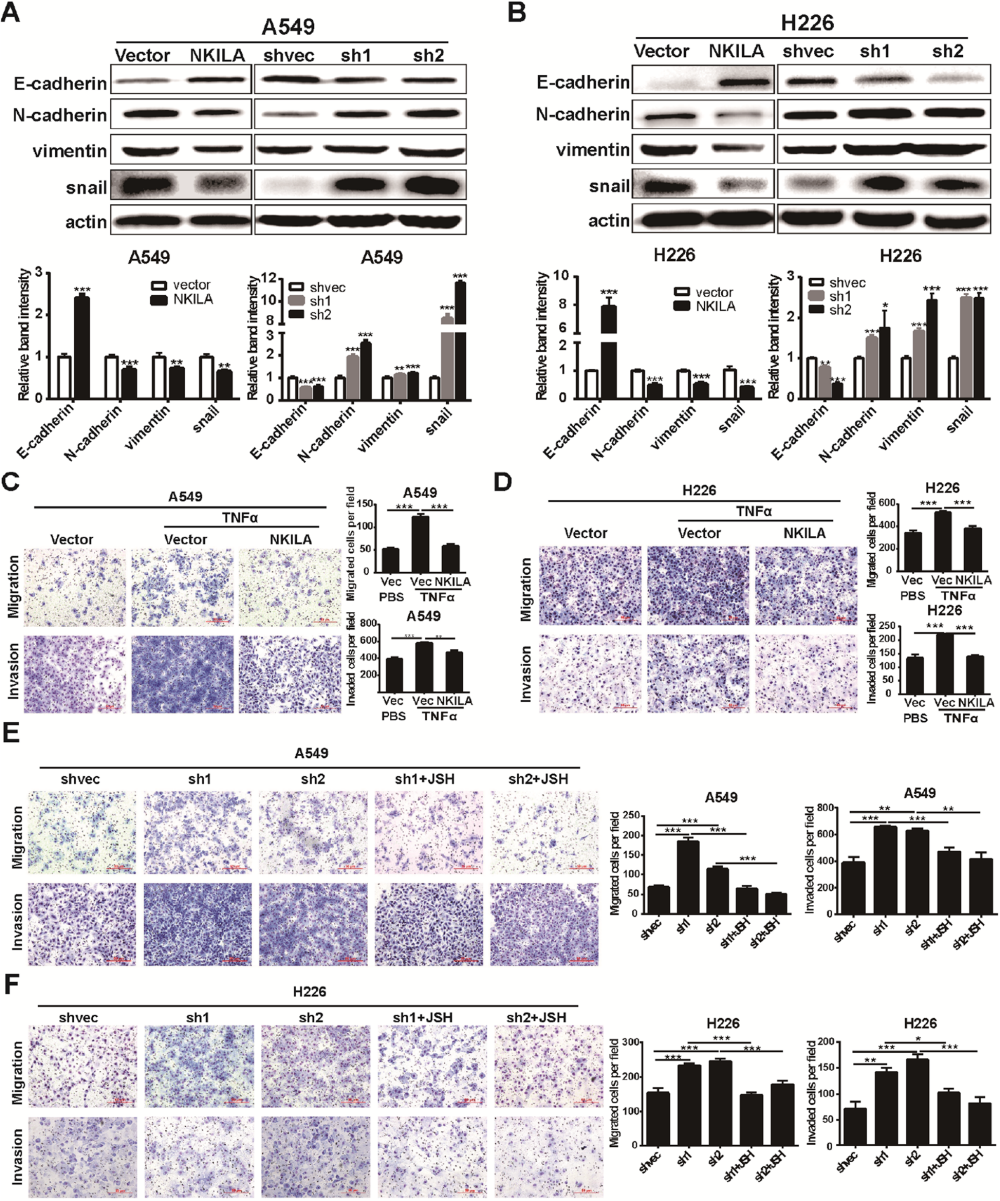
NKILA regulate NSCLC cell mobility via NF-κB/Snail pathway. **a** and **b** The expression levels of classical EMT markers in A549 (**a**) and H226 (**b**) cells were measured by western blot. **c** and **d** Migration and invasion ofA549 and H226 stably expressing NKILA or mock-vehicle control with or without TNFα stimuli measured by Chamber assay. (**e** and **f**) Migration and invasion of A549 and H226 stably expressing NKILA shRNA or negative control with or without JSH-23 (JSH) measured by Chamber assay. Left panel was representative images and *right panel* was statistical column diagram. Data are expressed as means ± SEM, *n* = 3. *p < 0.05, **p < 0.01, ***p < 0.001

The corrections do not compromise the validity of the conclusions and the overall content of the article. The original article [1] has been updated.
