# Supplementary material for: Long non-coding RNA NKILA inhibits migration and invasion of non-small cell lung cancer via NF-κB/Snail pathway
Source: J Exp Clin Cancer Res. 2017 Apr 17;36:54. doi: 10.1186/s13046-017-0518-0 (PMC5393036; doi:10.1186/s13046-017-0518-0)
Supplement: Supplementary file 1 — PMEPA1 was upregulated by TGF-β1. Western blot for PMEPA1 in A549 and H226 cells with or without TGF-β1 induce. GAPDH is the loading control. Figure S2. NKILA-regulated Snail/EMT pathway change can be abrogated by NF-κB inhibitor JSH-23. NKILA-knockdown cells were incubated in TNFα with or without NF-κB inhibitor JSH-23, and the expression levels of classical EMT markers and p-IκB were measured by western blot. GAPDH is the loading control. Figure S3. NF-κB regulated NKILA expression can be reversed by TGF-β inhibitor. NKILA expression levels of A549 (A) and H226 (B) treated with TGF-β1 with or without JSH-23 (JSH) as well as the NKILA expression levels of NSCLC cells treated with TNFα or IL1β with or without SB505124 (SB) were detected by qRT-PCR. Data are expressed as means ± SEM, n = 3. Two-tailed Student’s t-test was used. *p < 0.05, ***p < 0.001, ns means no statistical significance. (DOCX 566 kb) [file 13046_2017_518_MOESM1_ESM.docx]

**
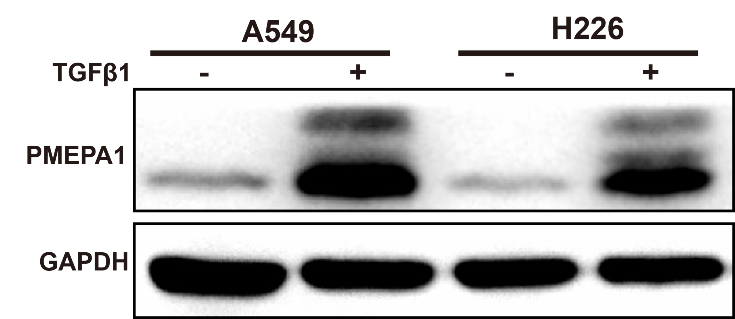
**

**Figure S1. PMEPA1 was upregulated by TGF-β1**. Western blot for PMEPA1 in A549 and H226 cells with or without TGF-β1 induce. GAPDH is the loading control.


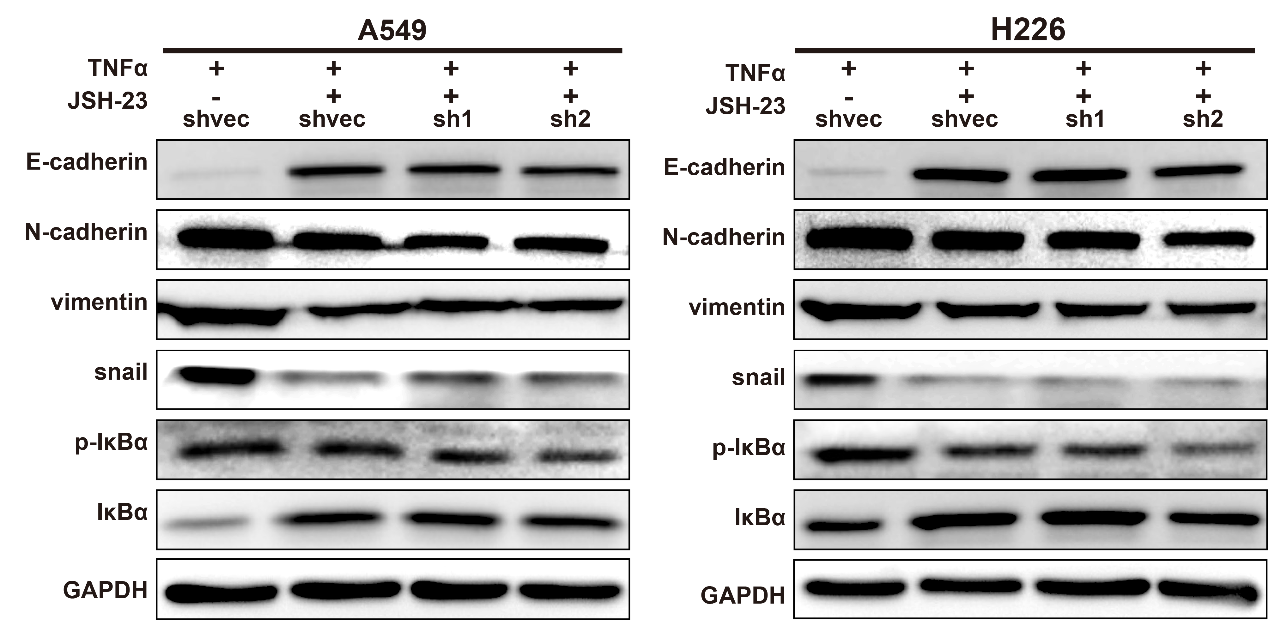


**Figure S2. NKILA-regulated Snail/EMT pathway change can be abrogated by NF-κB inhibitor JSH-23.** NKILA-knockdown cells were incubated in TNFα with or without NF-κB inhibitor JSH-23, and the expression levels of classical EMT markers and p-IκB were measured by western blot. GAPDH is the loading control.


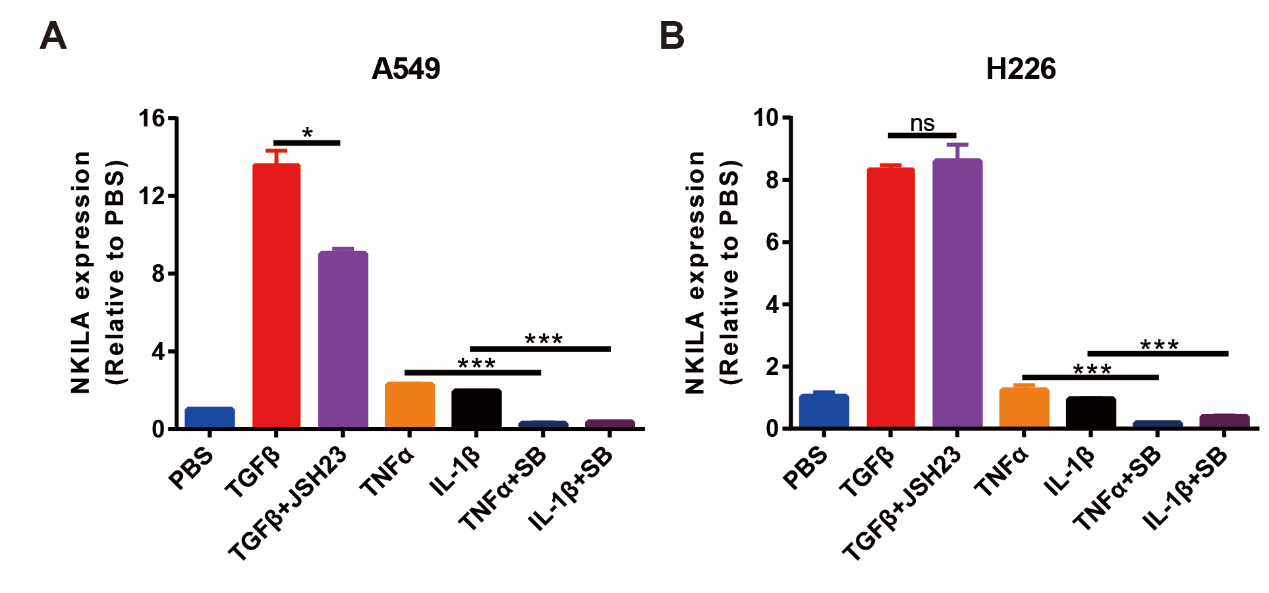


**Figure S3. NF-κB regulated NKILA expression can be reversed by TGF-β inhibitor.** NKILA expression levels of A549 (A) and H226 (B) treated with TGF-β1 with or without JSH-23 (JSH) as well as the NKILA expression levels of NSCLC cells treated with TNFα or IL1β with or without SB505124 (SB) were detected by qRT-PCR. Data are expressed as means ± SEM, n=3. Two-tailed Student’s t-test was used. *p < 0.05, ***p < 0.001, ns means no statistical significance.
