# Supplementary material for: Long non-coding RNA NKILA inhibits migration and invasion of non-small cell lung cancer via NF-κB/Snail pathway
Source: J Exp Clin Cancer Res. 2017 Apr 17;36:54. doi: 10.1186/s13046-017-0518-0 (PMC5393036; doi:10.1186/s13046-017-0518-0)
Supplement: Supplementary file 2 — JASPAR predicted Smad2/3 binding sites of NKILA promoter area. (DOCX 14 kb) [file 13046_2017_518_MOESM2_ESM.docx]

| **Model ID** | **Model name** | **Score** | **Relative score** | **Start** | **End** | **Strand** | **predicted site sequence** |
| --- | --- | --- | --- | --- | --- | --- | --- |
| MA0513.1 | SMAD2::SMAD3::SMAD4 | 7.770 | 0.812 | 1946 | 1934 | 1 | cagcctggccccc |
| MA0795.1 | SMAD3 | 4.826 | 0.804 | 1817 | 1808 | -1 | cttctagcct |
| MA0513.1 | SMAD2::SMAD3::SMAD4 | 7.534 | 0.808 | 1570 | 1558 | -1 | ctggctgagacca |
| MA0795.1 | SMAD3 | 11.065 | 0.900 | 1545 | 1536 | -1 | tgtctggaca |
| MA0795.1 | SMAD3 | 11.781 | 0.910 | 1545 | 1536 | 1 | tgtccagaca |
| MA0513.1 | SMAD2::SMAD3::SMAD4 | 7.365 | 0.805 | 1513 | 1501 | -1 | gtgtctgtgtccc |
| MA0513.1 | SMAD2::SMAD3::SMAD4 | 8.091 | 0.817 | 1241 | 1229 | -1 | ctgtctgggtctt |
| MA0795.1 | SMAD3 | 5.334 | 0.812 | 1121 | 1112 | 1 | agaccagaca |
| MA0513.1 | SMAD2::SMAD3::SMAD4 | 11.633 | 0.875 | 1111 | 1099 | 1 | gtgtctcacacgc |
| MA0513.1 | SMAD2::SMAD3::SMAD4 | 7.727 | 0.811 | 918 | 906 | 1 | caggctcgcagcg |
| MA0513.1 | SMAD2::SMAD3::SMAD4 | 7.813 | 0.812 | 190 | 178 | -1 | ctgcctgcctccg |
| MA0513.1 | SMAD2::SMAD3::SMAD4 | 8.714 | 0.827 | 186 | 174 | -1 | ccgtctgcctgcc |

**Table S1. JASPAR predicted Smad2/3 binding sites of NKILA promoter area**
